# Einschätzung des subjektiven Infektionsrisikos und Impfbereitschaft gegen SARS-CoV-2 unter deutschen Augenärzten: Ergebnisse einer Umfrage von DOG und BVA

**DOI:** 10.1007/s00347-021-01425-1

**Published:** 2021-05-21

**Authors:** M. Roth, C. Holtmann, A. Tillmann, B. Bertram, G. Geerling

**Affiliations:** 1 Klinik für Augenheilkunde, Universitätsklinikum Düsseldorf, Moorenstr. 5, 40225 Düsseldorf, Deutschland; 2 Augenarztpraxis Aachen, Aachen, Deutschland

**Keywords:** Umfrage, Infektionsrisiko, Corona, Impfbereitschaft, SARS-CoV‑2, Survey, Infection risk, Corona, Vaccine hesistancy, SARS-CoV‑2

## Abstract

**Hintergrund und Ziele:**

Nach Zulassung erster COVID-19-Impfstoffe in Deutschland sind „Impfpriorisierung“ und „Impfbereitschaft“ zentrale Themen in der Diskussion über Strategien zur Beendigung der Pandemie. Wie Augenärztinnen und Augenärzte das Infektionsrisiko in Augenkliniken und -praxen subjektiv bewerten und wie groß die Impfbereitschaft in dieser Berufsgruppe ist, wurde bisher nicht untersucht. Ziel dieses Projekts war die Erfassung der subjektiven Bewertung des Infektionsrisikos und der Impfbereitschaft der Augenärzte in Deutschland.

**Methoden:**

Die Daten wurden im Rahmen einer kurzen, anonymen Online-Umfrage des Berufsverbands der Augenärzte Deutschlands (BVA) und der Deutschen Ophthalmologischen Gesellschaft (DOG) unter Federführung der Universitätsaugenklinik Düsseldorf erhoben. Der Fragebogen war im Zeitraum vom 22.01. bis 12.02.2021 zur Teilnahme freigeschaltet. Die Umfrage richtete sich an alle augenärztlichen Kolleginnen und Kollegen.

**Ergebnisse:**

Insgesamt wurden 1162 vollständige Antwortbögen ausgewertet. Das berufsbedingte Infektionsrisiko bewerten die Befragten durchschnittlich mit 7,5 ± 1,9 (Skala von 1 bis 10; 1 = sehr geringes Risiko, 10 = sehr hohes Risiko); 971 Umfrageteilnehmer (83,6 %) schätzen das Infektionsrisiko im Vergleich zu anderen ärztlichen Fachrichtungen als höher ein; 92,9 % (*n* = 1079) der Umfrageteilnehmer geben an, sich impfen lassen zu wollen.

**Schlussfolgerung:**

Die befragten Augenärzte sehen ihre Berufsgruppe einem auch im Vergleich zu anderen Fachrichtungen überdurchschnittlich hohen SARS-CoV-2-Infektionsrisiko ausgesetzt. Dabei kritisieren sie häufig die Priorisierung des Bundesministeriums für Gesundheit (BMG), die von der Priorisierung der Ständigen Impfkommission (STIKO) abweicht. Die Impfbereitschaft ist unter den befragten deutschen Augenärzten sehr hoch.

## Abstract (en)

**Background and objective:**

After approval of the first COVID-19 vaccines in Germany, vaccination prioritization and vaccination preparedness are central topics in the discussion on strategies to end the pandemic. How ophthalmologists evaluate their risk of infection and whether they are willing to be vaccinated has not been investigated so far. The aim of this project was to assess the subjective rating of the risk of infection and the willingness to be vaccinated among German ophthalmologists.

**Methods:**

Data were collected by an anonymous online survey conducted by the Professional Association of Ophthalmologists in Germany (BVA) and the German Ophthalmological Society (DOG) under the auspices of the University Eye Hospital Düsseldorf. The questionnaire was open for participation from 22 January to 12 February 2021. The survey was addressed to all colleagues in ophthalmology.

**Results:**

A total of 1162 completed questionnaires were analyzed. On average, survey respondents rated their risk of infection as 7.5 ± 1.9 (scale of 1–10; 1 = very low risk, 10 = very high risk). Of the respondents 971 (83.6%) rated their risk of infection as higher compared to other disciplines and 92.9% (*n* = 1079) indicated they would be willing to be vaccinated.

**Conclusion:**

The ophthalmologists interviewed consider their professional group to be exposed to an above-average risk of SARS-COV‑2 infection compared to other disciplines. They frequently criticized the prioritization ranking of the German Ministry of Health (BMG), which deviated from the suggestions of the Standing Vaccination Committee of Germany (STIKO). The willingness to be vaccinated was very high among the surveyed German ophthalmologists.

## Einleitung und Fragestellung

Die durch „Severe-Acute-Respiratory-Syndrome-Coronavirus“ Typ 2 (SARS-CoV‑2) verursachte Pandemie stellt insbesondere Beschäftigte im Gesundheitswesen vor große Herausforderungen, da sie sich im Rahmen ihres Berufes häufig selbst infizieren und potenzielle Überträger der Erkrankung sein können [1, 2]. Augenärzte sind ebenso wie HNO- oder Zahnärzte bei der Untersuchung von Patienten aufgrund der nahezu stets unvermeidlichen Nähe zu den oberen Atemwegen einem erhöhten Infektionsrisiko ausgesetzt [3–7]. Hinzu kommen die Säuglinge und Kleinkinder bis zum Schulalter, die in der Regel keinen Mund-Nasen-Schutz bei der Untersuchung tragen.

Nach Empfehlung der Ständigen Impfkommission (STIKO) sollten Augenärzte demnach mit hoher Priorität geimpft werden, nämlich aus dem niedergelassenen Bereich zusammen nur mit Haus- und Kinderärzten und HNO- und Zahnärzten in der zweiten von insgesamt 6 Impf-Stufen (Stand Februar 2021) [6]. In der „Verordnung zum Anspruch auf Schutzimpfung gegen das Coronavirus SARS-CoV-2“ des Bundesministeriums für Gesundheit (BMG) (Stand: 08.02.2021) werden die Ärzte mit regelmäßigem, unmittelbarem Patientenkontakt aus allen Fachgruppen der zweiten Gruppe mit hoher Priorität zugeordnet. Da es allerdings nur noch 4 Gruppen gibt und die STIKO-Gruppen 2 und 3 weitgehend in die Gruppe 2 der Verordnung zusammengefasst werden, könnte sich der Impfzeitpunkt in dieser viel größeren Gruppe nach hinten verschieben. Damit wurde vom Gesetzgeber das Expositionsrisiko aller ärztlichen Fachrichtungen gleich bewertet [8]. Davon ausgenommen sind beispielsweise das direkt COVID-19-Erkrankte behandelnde Personal auf Intensivstationen und das Personal in Rettungsdiensten oder Impfzentren. Diese Ärzte wurden der ersten Gruppe mit der höchsten Priorität zugeordnet. Unklar ist bislang, wie Augenärztinnen und Augenärzte das Infektionsrisiko subjektiv bewerten und wie impfbereit sie sind. Vor diesem Hintergrund sollten deshalb mit einer kurzen, anonymen Umfrage des Berufsverbands der Augenärzte Deutschlands (BVA) und der Deutschen Ophthalmologischen Gesellschaft (DOG) unter Federführung der Universitätsaugenklinik Düsseldorf das subjektiv empfundene SARS-CoV-2-Infektionsrisiko sowie die Impfbereitschaft unter deutschen Augenärzten ermittelt werden.

## Methoden

In einem Online-Fragebogen (Lime Survey®, Hamburg, Deutschland) wurden Daten erhoben zu Arbeitssituation (Praxis/MVZ oder Klinik), Alter und Geschlecht, Vorliegen von Risikofaktoren, Schutzmaßnahmen im beruflichen Alltag, Einschätzung des Infektionsrisikos für den Augenarzt bzw. für den Patienten, sich mit SARS-CoV‑2 zu infizieren (Skala von 1 bis 10; 1 = sehr geringes Risiko, 5 und 6 = durchschnittliches Risiko, 10 = sehr hohes Risiko), Einschätzung des Infektionsrisikos für den Augenarzt bzw. für den Patienten im Vergleich zu anderen Fachrichtungen, Impfbereitschaft in der Vergangenheit (bezogen auf die Grippeschutzimpfung) sowie im Jahr 2021 (bezogen auf die SARS-CoV-19-Impfung) sowie der Zufriedenheit mit der Impfstrategie und Öffentlichkeitsarbeit der Bundesregierung (Freitext zur Begründung bei Unzufriedenheit wurde in entsprechend passende Kategorien eingeteilt, um eine Auswertung zu ermöglichen) zum Zeitpunkt der Befragung. Der komplette Fragebogen ist online zu diesem Beitrag einzusehen. Die Umfrage richtete sich an alle augenärztlichen Kolleginnen und Kollegen. In den elektronischen Rundschreiben des BVA am 22.01.2021 sowie der DOG vom 29.01.2021 wurde zur Teilnahme an der Umfrage aufgerufen. Die Umfrage war vom 22.01.2021 bis zum 12.02.2021 zur Teilnahme freigeschaltet.

### Statistische Auswertung

Die statistische Auswertung erfolgte mit Prism 9.0 (GraphPad, La Jolla, Californien, USA). Neben der deskriptiven Darstellung der Daten mit Mittelwert und Standardabweichung erfolgten bei nicht normalverteilten Variablen eine Korrelationsanalyse nach Spearman und Gruppenvergleiche mit dem Mann-Whitney-Test. Für die Untersuchung von Zusammenhängen nominaler und intervallskalierter Parameter wurde der Chi(χ^2^)-Test verwendet. Der Trend in den Grippeimpfungen im Verlauf von 2015 bis 2020 wurde mittels Cochrane Armitage Trend Test evaluiert. *p*-Werte ≤0,05 wurden als statistisch signifikant erachtet.

## Ergebnisse

Bis zum Abschluss am 12.02.2021 nahmen 1393 Augenärzte an der Umfrage teil; 231 unvollständige/abgebrochene Antwortbögen wurden von der Auswertung ausgeschlossen; 1162 vollständige Antwortbögen wurden ausgewertet. Das Durchschnittsalter dieser 1162 Umfrageteilnehmer lag bei 51 ± 11 Jahren; 58,3 % (*n* = 677) waren Frauen, 41,7 % (*n* = 484) Männer, 0,08 % (*n* = 1) divers; 87 % (*n* = 1016) der Umfrageteilnehmer gaben an, in einer Praxis zu arbeiten, 13 % (*n* = 146) in einer Klinik mit Bettenstation. Das Durchschnittsalter der Augenärzte in einer Praxis war signifikant höher als das in der Klinik (*p* < 0,001; Tab. 1).

**Table 1:** 

	Klinik (*n* = 146)	Praxis/MVZ (*n* = 1016)	*p*
*Alter*	42 ± 12 Jahre	53 ± 10 Jahre	*p* < 0,001
*Patienten/Tag*	26 ± 13	47 ± 16	*p* < 0,001
*Risiko für Arzt*	7,1 ± 2,0	7,5 ± 2,0	0,01
*Schutzmaßnahmen*			
– Regelmäßiges Lüften	71,9 %	93,3 %	<0,001
– Transp. Schutzschild an Spaltlampe	85,6 %	88,1 %	n. s.
– FFP-2-Maske	87,0 %	86,3 %	n. s.
– Schutzschild am Empfang	63,7 %	83,0 %	<0,001
– Verpfl. Händedesinfektion	65,1 %	78,8 %	<0,001
– Reduzierte Patienteneinbestellung	39,7 %	56,9 %	<0,001
– Einfacher MNS	28,8 %	17,7 %	0,01
– Temperaturkontrolle	30,1 %	12,3 %	<0,001
– * Raumluftaufbereitung	0,7 %	9,6 %	<0,001
– * FFP-3-Maske	1,4 %	7,4 %	0,01
– * Abstand	4,0 %	0	0,01
– * Mögl. keine Begleitpersonen	3,4 %	3,3 %	ns
– * Vermehrte Flächendesinfektion	2,1 %	3,2 %	ns
– * PCR/Schnelltests	6,8 %	1,2 %	<0,001
– * Symptomabfrage	6,2 %	0,7 %	<0,001
– * Red. Kontaktzeit	0	0,6 %	ns

Bei den Schutzmaßnahmen ist der prozentuale Anteil der Befragten aus der jeweiligen Gruppe angegeben, der die jeweilige Schutzmaßnahme genannt hat. Die mit * markierten Schutzmaßnahmen wurden im Freitext von den Befragten angegeben und in entsprechende Kategorien zusammengefasst

*FFP* partikelfiltrierende Halbmasken, englisch: „filtering face piece“, *MNS* Mund-Nasen-Schutz, *ns* nicht signifikant, *PCR* Polymerasekettenreaktion, englisch: „polymerase chain reaction“

### Risikofaktoren

**Figure 1:**
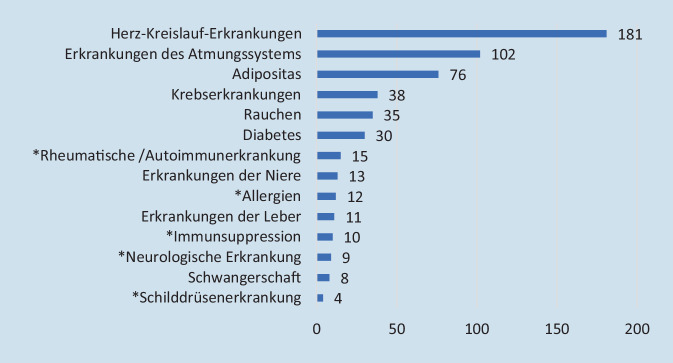

Insgesamt gaben 400 Umfrageteilnehmer (34,4 %) das Vorliegen möglicher Risikofaktoren bzw. Grunderkrankungen an, die das Infektionsrisiko an COVID 19 erhöhen. Dabei zeigte sich ein signifikanter Unterschied in der Alters- und Geschlechterverteilung (Tab. 2). Unter denjenigen Befragten, die Risikofaktoren angaben, lag der Anteil der Männer bei 52,6 %, unter denjenigen ohne Risikofaktoren bei 36 % (*p* < 0,001). Die Teilnehmer mit Risikofaktoren waren mit 55 ± 10 Jahren zudem signifikant älter als diejenigen ohne Risikofaktoren mit 49 ± 11 Jahren (*p* < 0,001). Die häufigsten angegebenen Grunderkrankungen waren Herz-Kreislauf-Erkrankungen (*n* = 181), Erkrankungen der Atemwege (*n* = 102) und Adipositas (*n* = 76) (Abb. 1).

**Table 2:** 

	Risikofaktoren JA (*n* = 400)	Risikofaktoren NEIN (*n* = 762)	*p*
*Alter*	55 ± 10 Jahre	49 ± 11 Jahre	<0,001
*Geschlechteranteil*	47,4 % weiblich 52,6 % männlich	64,0 % weiblich 36 % männlich	<0,001

### Schutzmaßnahmen

**Figure 2:**
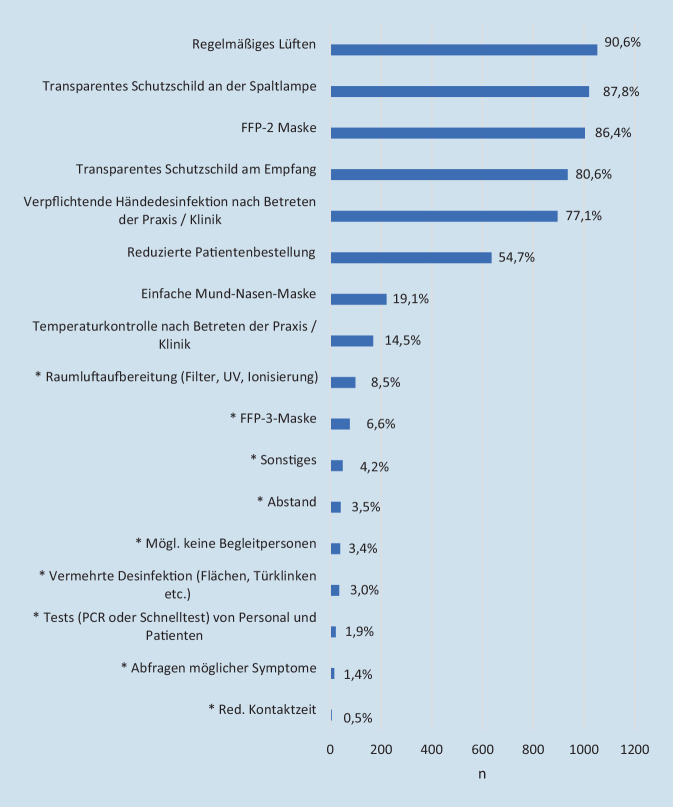

Die insgesamt am meisten angegebenen Schutzmaßnahmen waren regelmäßiges Lüften (90,6 %), ein transparentes Schutzschild an der Spaltlampe (87,8 %) und eine FFP-2-Maske (86,4 %); 54,7 % der Befragten gaben an, die Patientenzahl reduziert zu haben, und 8,5 %, Maßnahmen zur Raumluftaufbereitung installiert zu haben. Die vollständige Auflistung der Schutzmaßnahmen ist in Abb. 2 dargestellt. Zwischen Klinik und Praxis/MVZ zeigten sich Unterschiede bei einzelnen angegebenen Schutzmaßnahmen, die in Tab. 1 zusammengefasst sind.

### Risikobewertung

**Figure 3:**
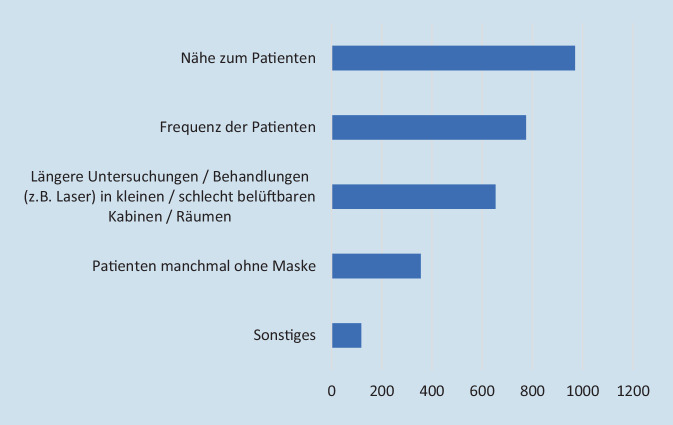

Das berufsbedingte SARS-CoV-2-Infektionsrisiko der Augenärzte bewerteten die Umfrageteilnehmer durchschnittlich mit 7,5 ± 1,9 (Skala von 1 bis 10; 1 = sehr geringes Risiko, 10 = sehr hohes Risiko). Das Risiko, dem Patienten beim Augenarzt ausgesetzt sind, wurde mit durchschnittlich 5,2 ± 2,2 bewertet (Skala von 1 bis 10; 1 = sehr geringes Risiko, 10 = sehr hohes Risiko). Die Einschätzung des persönlichen Risikos korrelierte deutlich mit der Einschätzung des Risikos für die Patienten (*p* < 0,001, ρ(1160) = 0,45); 971 Umfrageteilnehmer (83,6 %) gaben an, das Risiko, dem Augenärzte im Vergleich zu anderen Fachrichtungen ausgesetzt sind, als höher einzuschätzen. Diese Einschätzung war ausgeprägter bei Umfrageteilnehmern mit Risikofaktoren (*p* < 0,001). Das Risiko für den Patienten hingegen schätzen nur 33,9 % (*n* = 394) der Umfrageteilnehmer beim Augenarzt höher ein als in anderen Fachrichtungen. In beiden Fällen gaben die Umfrageteilnehmer v. a. die Nähe und die hohe Zahl der untersuchten Patienten als Grund der Risikobewertung an (Abb. 3 und 4).

**Figure 4:**
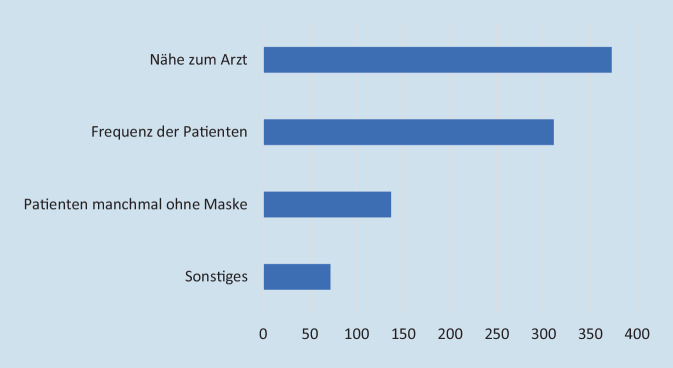

Die Zahl der untersuchten Patienten pro Tag lag bei durchschnittlich 44 ± 17 und unterschied sich signifikant zwischen Klinik und Praxis (*p* < 0,001). Die Zahl der Patienten pro Tag korrelierte mit der subjektiven Risikoeinschätzung, die von Teilnehmern der Umfrage sowohl für den Arzt als auch die Patienten angegeben wurde (Arzt: *p* < 0,001, ρ(1146) = 0,16; Patient: *p* = 0,02, ρ(1146) = 0,07). Augenärzte in der Klinik schätzten das berufsbedingte Infektionsrisiko signifikant niedriger ein als die in einer Praxis tätigen Kollegen (*p* = 0,01).

Mit zunehmendem Alter schätzten die Umfrageteilnehmer das Risiko für den Arzt höher ein (*p* = 0,01, ρ(1160) = 0,08). Die Risikoeinschätzung für den Patienten hingegen sank mit zunehmendem Alter der Augenärzte (*p* < 0,001, ρ(1160) = −0,19).

Umfrageteilnehmer, die angaben, sich impfen lassen zu wollen (s. unten), bewerteten das Risiko für sich und für Patienten signifikant höher (jeweils *p* < 0,001; Tab. 3).

**Table 3:** 

	Impfwillige (*n* = 1079)	Impfzögerer (*n* = 83)	*p*
*Risiko für Arzt*	7,7 ± 1,7	4,9 ± 3,0	<0,001
*Risiko für Patient*	5,3 ± 2,2	3,7 ± 2,4	<0,001

Das Risiko für Augenärzte im Vergleich zu anderen Fachrichtungen wurde von den befragten Impfzögerern (von engl. „vaccine hesistancy“) signifikant weniger häufig als „hoch“ angegeben (*p* < 0,001).

Es hatten 54,4 % der Umfrageteilnehmer (*n* = 632) Sorge, dass eine Mutation die Wirksamkeit der Impfung einschränken könnte. Die Umfrageteilnehmer, die bezüglich der Mutationen besorgt waren, waren mit 50 ± 11 Jahren signifikant jünger als diejenigen, die keine Sorge vor Mutationen hatten (52 ± 11 Jahre) (*p* = 0,01).

### Impfbereitschaft

**Figure 5:**
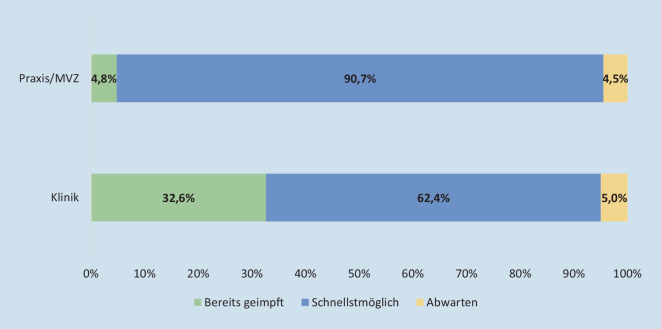

Insgesamt geben 92,9 % (*n* = 1079) der Umfrageteilnehmer an, sich impfen lassen zu wollen, und 90,4 % (*n* = 1051) bzw. 89,7 % (*n* = 1042), dass sich auch möglichst alle ärztlichen bzw. nichtärztlichen Mitarbeiter impfen lassen sollten; 87 % (*n* = 936) der Umfrageteilnehmer wollten sich so schnell wie möglich impfen lassen. Lediglich 8,5 % (*n* = 91) waren zu diesem Zeitpunkt schon geimpft; 3,5 % (*n* = 49) wollten zunächst noch abwarten. Die Teilnehmer, die sich möglichst schnell impfen lassen wollten, waren mit 52 ± 11 Jahren signifikant älter als die, die sich impfen lassen, aber noch abwarten wollten (48 ± 12 Jahre). Die bereits geimpften Teilnehmer stellten mit einem Durchschnittsalter von 45 ± 13 Jahren nicht nur die jüngste Kohorte dar, sondern waren überproportional häufig Klinikmitarbeiter (*p* < 0,001) (Abb. 5).

Nur 7,1 % (*n* = 83) gaben an, sich nicht impfen lassen zu wollen. Darunter gaben 53 Personen als Grund eine Unsicherheit bezüglich der Impfstoffe an, 8 eine Neigung zu anaphylaktischer Reaktion; 6 bereits eine SARS-CoV-2-Infektion durchgemacht zu haben, und 16 gaben „Sonstiges“ an. Die Bereitschaft zur bzw. Ablehnung der Impfung zeigte keine Abhängigkeit/Zusammenhang mit Geschlecht, Alter, Arbeitsplatz (Praxis/Klinik) oder dem Vorliegen von Risikofaktoren bzw. Grunderkrankungen. Bei 34 der 83 Befragten (40,9 %), die sich nicht impfen lassen möchten, lagen Risikofaktoren bzw. Grunderkrankungen vor und damit tendenziell häufiger als unter Impfwilligen (33,9 %; ns).

**Figure 6:**
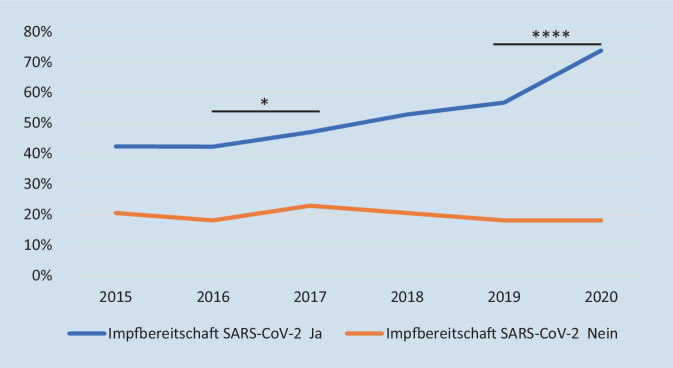

In allen abgefragten Jahren (2015 bis 2020) zeigte sich ein hochsignifikanter Zusammenhang zwischen der Impfbereitschaft und einer absolvierten Grippeschutzimpfung (*p* < 0,0001). Insgesamt und bei den impfwilligen Umfrageteilnehmern hat die Zahl der Grippeschutzimpfungen über die Jahre signifikant zugenommen (Cochrane Armitage Trend Test *p* < 0,0001), nicht aber bei den Impfzögerern. Im Vergleich jeweils zweier aufeinanderfolgender Jahre zeigt sich lediglich für 2016 bis 2017 eine signifikante (*p* = 0,03) und für 2019 bis 2020 eine hochsignifikante Zunahme der Grippeschutzimpfungen (*p* < 0,0001) (Abb. 6).

### Einschätzung der Impfstrategie und der öffentlichen Aufklärung zur Impfung

**Figure 7:**
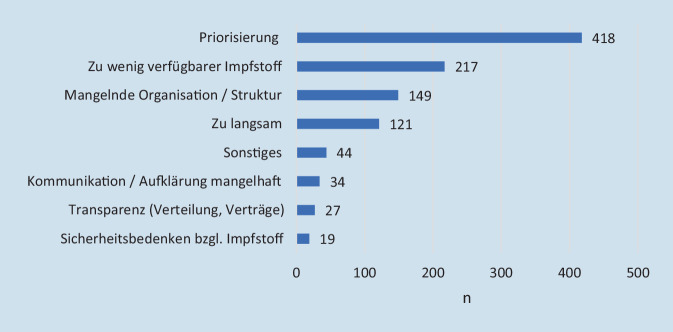

Ungefähr zwei Drittel der Umfrageteilnehmer äußerten Unzufriedenheit mit der Impfstrategie der Bundesregierung (*n* = 814; 70,1 %). Die häufigsten angegebenen Kritikpunkte bezogen sich auf die Priorisierung (*n* = 418), auf zu wenig bzw. zu spät bestellten Impfstoff (*n* = 217), Defizite bei der Planung und Umsetzung der Impfungen (*n* = 149), die mangelnde Geschwindigkeit der Impfungen (*n* = 121) (weitere Angaben s. Abb. 7). Mit der nationalen Impfstrategie zufriedene Teilnehmer waren signifikant jünger (50 ± 11 Jahre), als die unzufriedenen (52 ± 11 Jahre) (*p* = 0,01).

**Figure 8:**
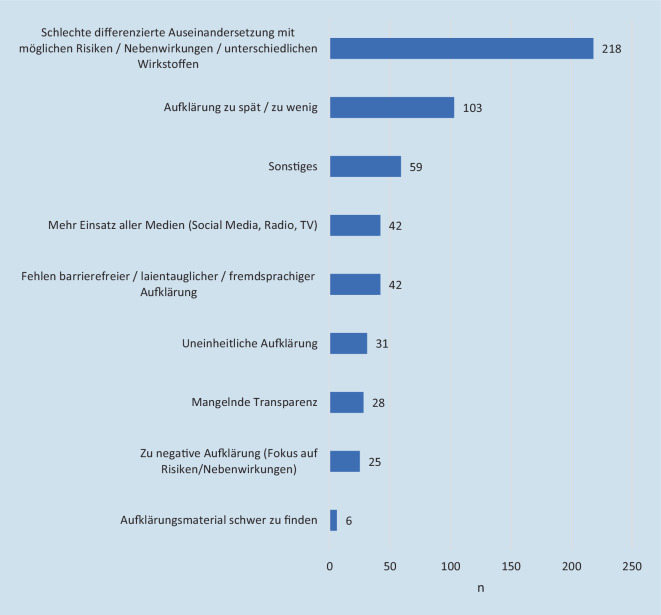

Die öffentliche Aufklärung zur Impfung wurde mit 53,4 % (*n* = 620) etwas positiver beurteilt. Am häufigsten wurden Mängel bezüglich möglicher Risiken/Nebenwirkungen, verschiedener Wirkstoffe und den zugrunde liegenden Wirkmechanismen und damit die Gefahr der Verunsicherung durch Falschinformationen kritisiert (*n* = 218); 103 Umfrageteilnehmer bewerteten die Aufklärungsarbeit als verspätet und inhaltlich ungenügend. Jeweils 42 Umfrageteilnehmer wünschten sich einen stärkeren Einsatz verschiedener Medien (TV, Radio, Social Media), barrierefreies und laientaugliches Informationsmaterial in verschiedenen Sprachen (Abb. 8).

## Diskussion

Laut Empfehlung der Ständigen Impfkommission (STIKO) sind „Personen, die berufsbedingt enge Kontakte mit anderen Menschen nicht vermeiden können, einem erhöhten Expositionsrisiko ausgesetzt“ [6]. „Aufgrund der engen Kontakte und dokumentierten Infektionsfällen bei medizinischem Personal“ wird das Expositionsrisiko für Augenärzte somit als hoch bewertet [6]. Diese Risikobewertung spiegelt sich auch in der subjektiven Einschätzung der befragten Augenärzte selber wider. Die Zahl der Augenärzte, die den Fragebogen vollständig ausgefüllt haben, entspricht jeweils einem Anteil von ca. 17 % der Empfänger des DOG-Newsletter und der Rundmail des BVA. Es haben ca. 13 % aller ca. 9200 Augenärzte und augenärztlichen Weiterbildungsärzte in deutschen Augenkliniken und -praxen/MVZ teilgenommen. Die Umfrageteilnehmer bewerteten das Risiko für Augenärzte auf einer Skala von 1 bis 10 überdurchschnittlich hoch und schätzten es auch mehrheitlich höher als in anderen Fachrichtungen ein.

Aufgrund der bisher schlechten Datenlage wurde im Rahmen der Diskussion teilweise auf noch nicht begutachtete Studien von sog. Preprint-Servern, Vorabergebnisse und Daten von Websites und Marktforschungsunternehmen zurückgegriffen.

### Impfbereitschaft in der Bevölkerung

Innerhalb der deutschen Bevölkerung liegt die Bereitschaft, sich gegen SARS-CoV‑2 impfen zu lassen, zwischen 53 und 79 %, nimmt nach einer Auswertung des Imperial College London zwischen November 2020 und Januar 2021 aber tendenziell zu [9–15]. Die Ablehnungsquote lag im April des Jahres 2020 in Deutschland bei 10 % [10]. Bezüglich möglicher Einflussfaktoren auf die Impfbereitschaft unterscheiden sich die zur Verfügung stehenden Angaben teilweise. Eine Onlinebefragung der Universitätsmedizin Mainz im Dezember 2020 und Januar 2021 zeigte eine höhere Impfbereitschaft unter Frauen und jüngeren Befragten sowie keinen Einfluss durch das Vorliegen einer chronischen Erkrankung [14]. Im Gegensatz dazu ist die Impfbereitschaft in einer Umfrage unter 800 thüringischen Einwohnern unter Männern höher [15]. Auch in Umfragen in der französischen Bevölkerung zeigte sich eine höhere Impfbereitschaft unter Männern, außerdem unter älteren Befragten, bei subjektiv höherer Risikoeinschätzung und höherem Bildungsniveau [16, 17].

### Impfbereitschaft im Gesundheitswesen

Die Ergebnisse einer Umfrage unter französischen Erwachsenen von Detoc et al. zeigten zwar bereits, dass auch die Arbeit im Gesundheitswesen mit einer höheren Impfbereitschaft einhergeht [17]. Die Daten zur Impfbereitschaft im Gesundheitswesen sind aber noch sehr begrenzt. Studien aus China und der Türkei berichten von einer Bereitschaft zur SARS-CoV-2-Impfung unter Angestellten im Gesundheitswesen von ca. 70 % [18, 19] und in Frankreich von 76,9 % [20]. Der Anteil der impfbereiten Augenärzte in unserer Studie ist somit vergleichsweise hoch.

Wie in der Allgemeinbevölkerung (s. oben) wird in Publikationen aus Israel, der Türkei, USA und China eine Abhängigkeit der Impfbereitschaft bei Angestellten im Gesundheitswesen von Alter und Geschlecht beschrieben, außerdem aber auch von der Berufsgruppe (Ärzte, Pflege, Auszubildende, Sonstige), von der jeweiligen medizinischen Fachrichtung und von der direkten Arbeit mit COVID-19-positiven Patienten oder der subjektiven Risikobewertung [18–22]. Tendenziell würden sich in Umfragen unter Angestellten im Gesundheitssektor Männer, ältere Personen und Personen, die das persönliche Risiko z. B. aufgrund entsprechender Kontakte zu Corona-Patienten höher bewerten, eher impfen lassen [20, 21, 23]. Im Gegensatz dazu zeigte sich in unserer Umfrage kein Zusammenhang zwischen Geschlecht, Alter oder dem Vorliegen von Risikofaktoren und der Bereitschaft bzw. Ablehnung der Impfung.

Eine Studie der Technischen Universität München zur Fach- und Personalgruppen-übergreifenden Impfbereitschaft und möglichen Einflussfaktoren bei medizinischem Personal hat die Datenerhebung am 28.02.2021 abgeschlossen. Die Ergebnisse stehen noch aus (www.covid-evidenz.de).

### Grippeimpfungen als prädiktiver Parameter für Akzeptanz der SARS-CoV-2-Impfung

Wie auch in unserer Studie ist die Bereitschaft, sich gegen Influenza impfen zu lassen, in mehreren Studien ein wichtiger prädiktiver Parameter für eine Akzeptanz der Impfung gegen SARS-CoV‑2 [19–21, 23, 24]. Der signifikante Anstieg der Grippeimpfungen von 2016 auf 2017 ist möglicherweise durch die in der Saison 2017 bis 2018 besonders stark grassierende Grippewelle erklärbar. Die hoch signifikante Zunahme der Grippeschutzimpfungen von 2019 auf 2020 führen wir auf die gesteigerte Impfbereitschaft im Rahmen der Corona-Pandemie zurückführen, nicht zuletzt wurde vom Bundesgesundheitsministerium vermehrt dazu aufgefordert [25]. In Umfragen aus Korea und Frankreich zeigte sich im gleichen Zeitraum jedoch kein Anstieg in der Akzeptanz der Grippeschutzimpfung [20, 23].

### Zufriedenheit der Gesellschaft mit der Impfkampagne

Zur Zufriedenheit der Gesellschaft mit der Impfkampagne in Deutschland gibt es wenig vergleichbare Daten. In der thüringischen COSMO-Studie ist das Vertrauen in die Bundesregierung von Oktober 2020 von 4,11 auf einen mittelmäßigen Wert von 3,77 im Januar 2021 gesunken (1 = sehr wenig Vertrauen; 7 = sehr viel Vertrauen) [15]. Eine Umfrage eines internationalen Marktforschungsunternehmens im Januar 2021 gibt die Unzufriedenheit über die Impfkampagne in der deutschen Bevölkerung mit 51 % und somit etwas niedriger als in unserer Umfrage an [12]. Der größte Kritikpunkt in unserer Umfrage war die Priorisierung der Impfung. Besonders häufig wurde eine höhere Priorisierung der medizinischen Berufe vor dem Alter gefordert. Auch in der Onlinebefragung der Universitätsmedizin Mainz wurde Alter gegenüber der Zugehörigkeit zu einem medizinischen Beruf nachrangig bewertet [14].

## Schlussfolgerung

Die befragten Augenärzte bewerten das subjektive Infektionsrisiko überdurchschnittlich hoch und kritisieren häufig die Priorisierung des BMG, die von der Priorisierung der STIKO abweicht. Die Bereitschaft, sich gegen SARS-CoV‑2 impfen zu lassen, ist unter deutschen Augenärzten sehr hoch. Die generelle Impfbereitschaft scheint in Infektionswellen vermehrt zu sein. Das Wissen um die Impfbereitschaft und die Risikoeinschätzung der Augenärzte ist nicht nur für diese, sondern auch für mögliche zukünftige Pandemien wichtig.
